# Nidulin stimulates glucose uptake in myotubes through the IRS-AKT pathway and alters redox balance and intracellular calcium

**DOI:** 10.1007/s13659-025-00546-3

**Published:** 2025-09-11

**Authors:** Kanittha Chantarasakha, Arunrat Yangchum, Masahiko Isaka, Surapun Tepaamorndech

**Affiliations:** 1 National Center for Genetic Engineering and Biotechnology (BIOTEC), National Science and Technology Development Agency (NSTDA), 111 Thailand Science Park, Phahonyothin Road, Klong Luang, Pathum Thani 12120 Thailand; 2 Department of Microbiology, Faculty of Medicine, Chulalongkorn University and King Chulalongkorn Memorial Hospital, Bangkok, 10330 Thailand

**Keywords:** Fungal metabolite, *Aspergillus*, Antidiabetic agent, Insulin resistance, Insulin signaling, GLUT4

## Abstract

Nidulin is a secondary metabolite of the depsidone family produced by *Aspergillus* spp., and has shown promises in pharmacological applications. This study aimed to investigate the effect of nidulin on glucose metabolism in skeletal muscle, the primary site of physiological glucose disposal, and its underlying mechanisms. Using a 2-[^3^H]-deoxy-glucose (2-DG) uptake assay, nidulin stimulated 2-DG in L6 myotubes in a dose- and time-dependent manner. This effect of nidulin was additive to insulin and metformin, and remained effective under palmitic acid-induced insulin resistance. At the molecular level, nidulin upregulated the mRNA expression and promoted membrane translocation of glucose transporters, GLUT4 and GLUT1. Although nidulin activated AMPK and p38 signaling, pharmacological inhibition of this pathway had minimal effect on nidulin-enhanced 2-DG uptake activity. Notably, nidulin activated key insulin signaling proteins, including IRS1, AKT, and p44/42, and its effect was attenuated by an AKT inhibitor. This study further compared the upstream mechanism of nidulin with that of insulin. While nidulin did not directly activate the insulin receptor β-subunit, it modulated redox homeostasis and intracellular calcium, evidenced by increased cytosolic H₂O₂ and Ca^2^⁺ levels. The 2-DG uptake-enhancing effect of nidulin and its activation of AKT were suppressed by either an antioxidant or calcium chelator treatment. These findings position nidulin as a promising insulin-sensitizing agent, offering mechanistic insights and therapeutic potential for improving glucose homeostasis in type 2 diabetes.

**Graphical Abstract:**

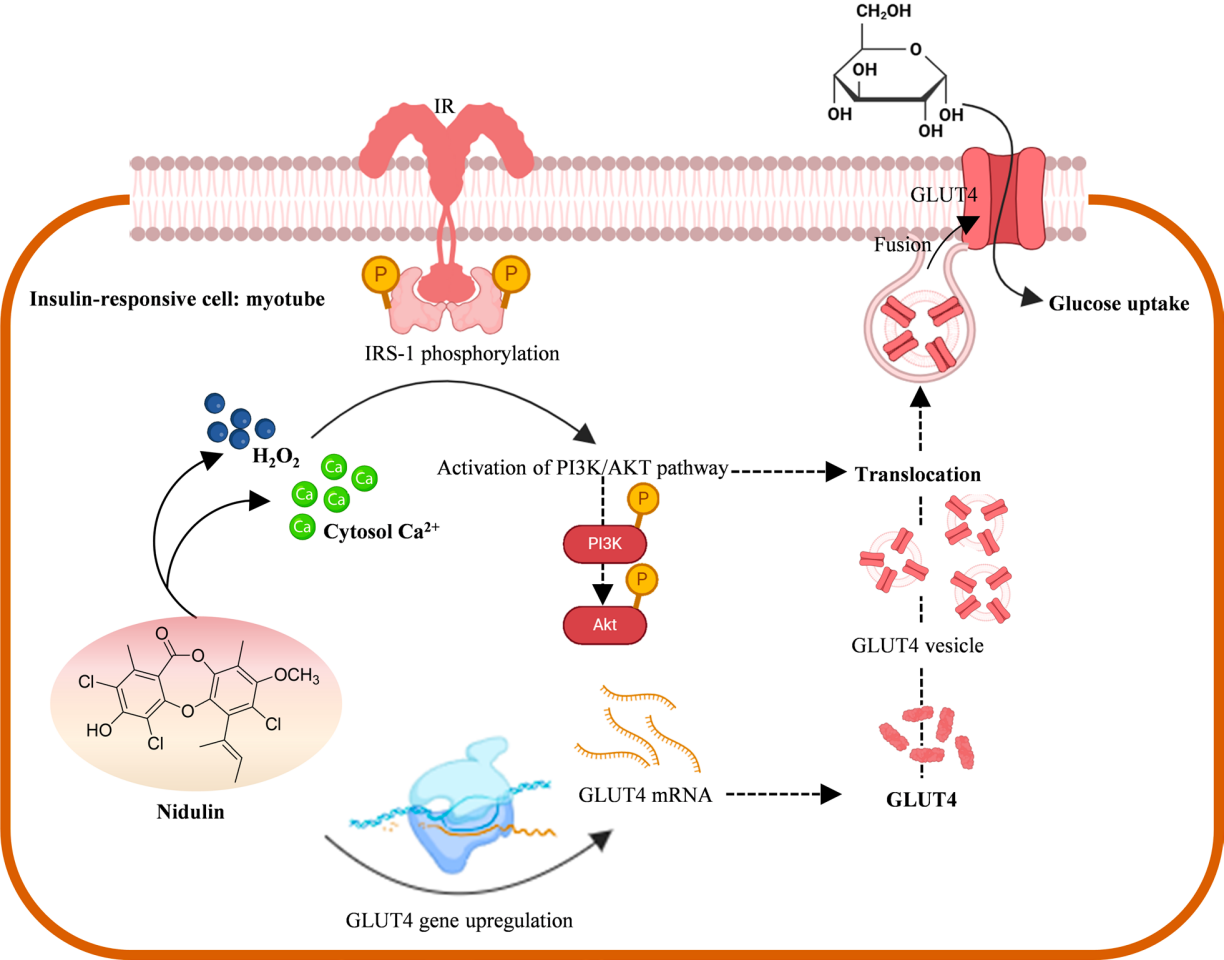

**Supplementary Information:**

The online version contains supplementary material available at 10.1007/s13659-025-00546-3.

## Introduction

Type 2 diabetes (T2D) is a chronic metabolic disorder characterized by insulin resistance and impaired insulin secretion, leading to persistent hyperglycemia [1]. The disease represents a significant global health challenge, and imposes a substantial economic burden. Despite advances in T2D treatment, current pharmacological options face several limitations. Conventional drugs, including insulin sensitizers and sodium-glucose cotransporter-2 inhibitors, are often associated with hypoglycemia, weight gain, and cardiovascular risks [2]. In addition, metformin which is regarded as the first-line treatment for T2D, has been linked to vitamin B12 deficiency in long-term use [3]. Importantly, metformin alone may not be sufficient to achieve glycemic control in patients with advanced T2D, [4, 5]. These challenges highlight the need for novel therapeutic agents with improved efficacy and safety profiles to address the unmet medical needs in T2D management.

Skeletal muscle is the primary tissue responsible for insulin-stimulated glucose disposal [6, 7]. Under normal physiological conditions, glucose uptake in skeletal muscle cells, such as myotubes, is regulated through the insulin-dependent pathway [6]. In response to postprandial glucose, pancreatic β-cell-secreted insulin binds to its receptor (IR), initiating a signaling cascade that involves the phosphorylation of insulin receptor substrate (IRS) proteins [8]. This leads to downstream phosphorylation of protein kinase B (AKT), which promotes the translocation of glucose transporter (GLUT) 4 to the plasma membrane, facilitating glucose uptake. Insulin also mediates redox sensing, including intracellular H₂O₂ and Ca^2^⁺ distribution, which activates downstream effectors and supports GLUT4 translocation [9, 10]. Alternatively, during energy stress, such as exercise or caloric restriction, glucose is taken up into skeletal muscle via the AMPK-dependent pathway [11]. AMPK functions as a cellular energy sensor, and its activation is independent of insulin. Once activated, AMPK promotes GLUT4 translocation to the plasma membrane, enhancing glucose uptake. The AMPK-dependent pathway is particularly relevant in conditions like insulin resistance, where insulin signaling is impaired, as it provides an alternative route for glucose transport [12]. Targeting these pathways has been a significant focus for developing therapeutic agents for T2D, especially those capable of improving glucose uptake in insulin-resistant states.

Natural products have emerged as promising sources for T2D treatment. Acarbose, an α-glucosidase inhibitor derived from fungi, has been successfully developed as a therapeutic agent for managing T2D. In the ongoing search for novel antidiabetic agents, fungus-derived compounds, including polysaccharides, sterols, and unidentified metabolites, have demonstrated significant potential [13–15]. Our research has previously identified a group of depsidones, secondary metabolites from *Aspergillus unguis*, as potent activators of glucose uptake in adipocytes [16]. Additionally, we found that a semi-synthetic depsidone, pyridylnidulin, ameliorated hyperglycemia and reduced hepatic steatosis in high-fat diet-induced obesity mice [17]. These findings highlight the untapped potential of fungal depsidones as therapeutic agents for T2D, targeting critical metabolic pathways to improve glucose homeostasis.

Among a group of depsidones with glucose uptake activity, nidulin contains the promising antidiabetic potential. However, its effects on skeletal muscle, which is a primary tissue responsible for whole-body glycemic control under normal physiological conditions, and is a critical site for the development of insulin resistance remained unexplored. Furthermore, the mechanisms underlying nidulin-stimulated glucose uptake were not fully understood. In this study, we utilized differentiated L6 myotubes to evaluate the glucose uptake stimulated by nidulin in both insulin-sensitive and insulin-resistant states. We also investigated the contribution of nidulin in intracellular glucose uptake signaling pathways.

## Results and discussion

### Nidulin activates glucose uptake in myotubes in a dose- and time-dependent manner

**Fig. 1:**
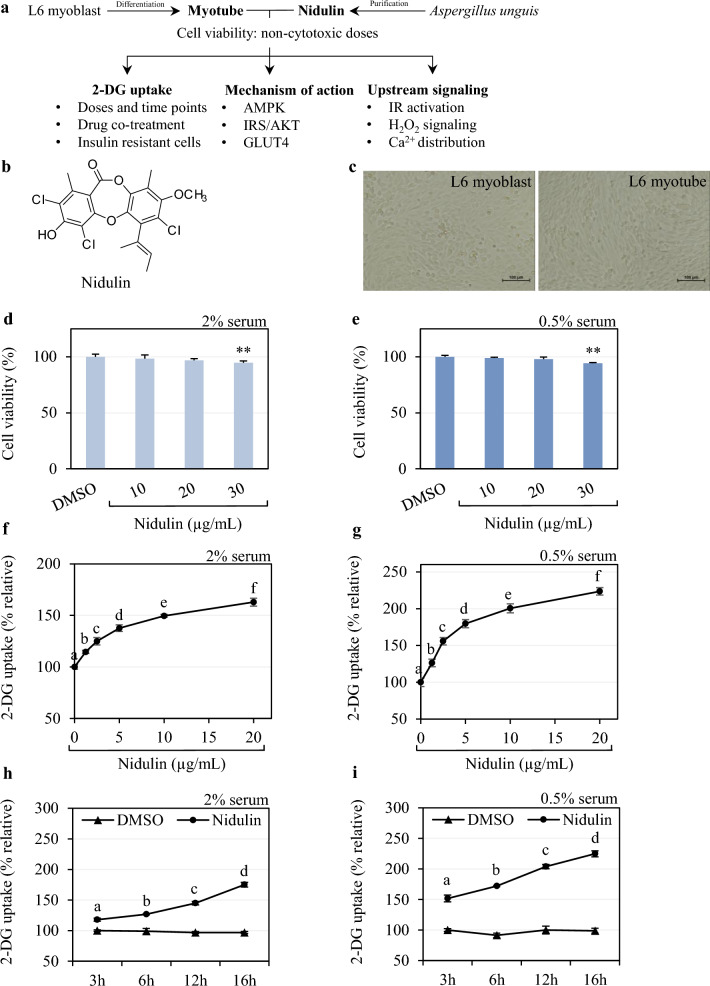
Effect of nidulin on 2-DG uptake in L6 myotubes. **a** Schematic diagram of the study. L6 myoblasts were differentiated to myotubes. The cells were treated with nidulin purified from *A. unguis* to determine the non-cytotoxic levels of nidulin. The effect of nidulin on 2-DG uptake and its mechanisms of action were investigated in the present study. **b** The structure of nidulin. **c** Representative microscope images (200 × total magnification) of L6 myoblasts and myotubes. **d** and **e** The number of viable L6 myotubes after 10, 20, and 30 µg/mL nidulin treatment for 18 h in differentiation medium containing 2% and 0.5% horse serum (HS), respectively. **f** and **g** Dose-dependent effects of nidulin on 2-DG uptake in differentiation medium containing 2% and 0.5% HS, respectively. Cells were treated with 1.25, 2.5, 5, 10, and 20 µg/mL nidulin for 16 h. **h** and **i** Time-dependent effects of nidulin (20 µg/mL) on 2-DG uptake in medium with 2% and 0.5% HS, respectively. Cells were treated for 3, 6, 12, and 16 h. Following treatment, a 2-DG uptake assay was performed. DMSO served as the vehicle control. Data are presented as relative values (%) and expressed as mean ± S.D. (n = 4 per group). Similar results were obtained from the multiple experiments. Significant differences compared to corresponding controls: **p < 0.01. Different letters denote statistically significant differences (*p* < 0.05)

The experimental design and nidulin structure are illustrated in Fig. 1a, b, respectively. L6 myoblasts were differentiated using horse serum (HS) to myotubes before use in this study (Fig. 1c). As shown in Fig. 1d, e, a non-cytotoxic concentration of nidulin was found ≤ 20 µg/mL in differentiation medium (2% HS) and low-serum medium (0.5% HS), respectively. In differentiation medium, treatment with 1.25, 2.5, 5, 10, and 20 µg/mL nidulin for 16 h significantly increased 2-DG uptake by 115, 125, 138, 150, and 163%, respectively, compared to untreated controls (Fig. 1f). Under low-serum conditions, the effect was more pronounced, with increases of 126, 156, 180, 200, and 224% at the same concentrations (Fig. 1g). The time-dependent effects of nidulin on 2-DG uptake were also examined under both serum conditions. In differentiation medium, treatment with 20 µg/mL nidulin increased 2-DG uptake by 118, 127, 145, and 175% after 3, 6, 12, and 16 h, respectively (Fig. 1h). In low-serum medium, uptake was elevated by 152, 172, 204, and 225% at the corresponding time points (Fig. 1i).

Nidulin, a trichlorinated depsidone-type secondary metabolite produced by *A. unguinol* in this study, has also been reported in *A. nidulans* [18, 19]. Like many fungal secondary metabolites, depsidones are believed to play a role in fungal defense and stress adaptation [20]. In humans, depsidones have demonstrated a range of promising pharmacological activities, including antimicrobial, anticancer, and antidiabetic effects [21]. Our previous work demonstrated that *Aspergillus*-derived depsidones promoted glucose uptake in differentiated 3T3-L1 adipocytes, with nidulin displaying the most potent effect among the compounds tested [16]. However, since skeletal muscle is the primary site of insulin-mediated glucose disposal, and insulin resistance in this tissue often precedes the onset of systemic T2D [22, 23], it is essential to determine whether nidulin can similarly enhance glucose uptake in myotubes. In these conditions, EC50 of nidulin in glucose uptake activity was 3.5 and 3.2 µg/mL in 2% and 0.5% HS, respectively. Accordingly, our results demonstrate that nidulin enhances glucose uptake in L6 myotubes in both a dose- and time-dependent manner, and its efficacy is maintained under serum-restricted conditions.

### Nidulin enhances glucose uptake during co-treatment with insulin and metformin, and in an insulin-resistant state

**Fig. 2:**
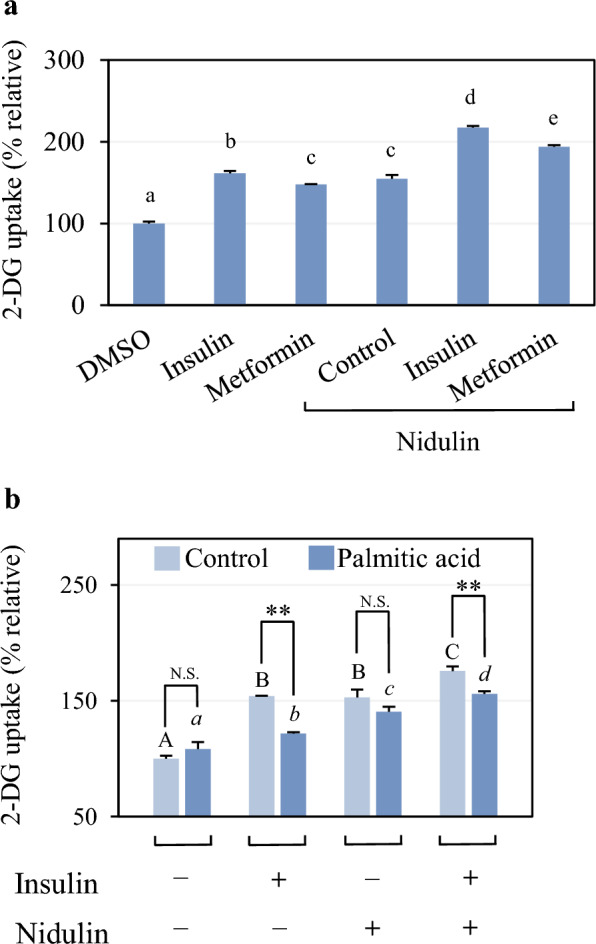
Effect of nidulin during drug treatment and under insulin resistance. **a** 2-DG uptake in the presence of insulin or metformin. L6 myotubes were treated with 20 µg/mL nidulin for 16 h, followed by 100 nM insulin treatment for 30 min, or co-treated with 1 mM metformin and 20 µg/mL nidulin for 16 h. A 2-DG uptake assay was performed after treatment. DMSO served as the vehicle control. **b** 2-DG uptake in palmitic acid-induced insulin resistance. Cells were treated with 125 µM palmitic acid and/or 20 µg/mL nidulin as indicated for 16 h. Vehicle controls were 50% EtOH (v/v) for palmitic acid and DMSO for nidulin. Where indicated, cells were stimulated with 100 nM insulin for 30 min prior to 2-DG uptake assay. Data are presented as relative 2-DG uptake (%) and expressed as mean ± S.D. (n = 4 per group). Similar results were obtained from the multiple experiments. Comparisons between the control group (no palmitate) and the palmitic acid-treated group were made. N.S., not significant; **p < 0.01. Comparisons within the control and palmitate-treated groups are indicated by capital and italic letters, respectively. Different letters denote statistically significant differences (*p* < 0.05)

As shown in Fig. 2a, treatment with 100 nM insulin, 1 mM metformin, and 20 µg/mL nidulin significantly increased 2-DG uptake by 162, 148, and 155%, respectively, compared to the untreated control. Co-treatment of nidulin with either insulin or metformin further elevated glucose uptake, showing an approximate 130% increase relative to each compound alone. These findings indicate that nidulin does not interfere with, but rather potentiates, insulin- and metformin-stimulated glucose uptake, suggesting an additive effect.

The effect of nidulin on 2-DG uptake was further assessed under insulin-resistant conditions induced by palmitic acid. As shown in Fig. 2b, in the absence of insulin or nidulin, intracellular 2-DG uptake levels were comparable between untreated control and palmitic acid-treated cells, confirming that basal uptake was not altered by palmitate. Insulin stimulation Markedly increased 2-DG uptake in control cells to 154%, while only reaching 121% in the palmitic acid-treated group. Consistent with previous findings, palmitate-treated L6 myotubes in our study exhibited a decrease in insulin-stimulated 2-DG uptake, a hallmark of insulin-resistant muscle cells [24]. Treatment with nidulin alone elevated 2-DG uptake to 153% in the control group and 141% in the insulin-resistant group. The lesser difference between these groups suggests that nidulin-stimulated glucose uptake is relatively insensitive to insulin resistance. Moreover, combined treatment with insulin and nidulin further enhanced 2-DG uptake to 176% in control cells and 156% in palmitate-treated cells. This increase (156%) closely matched the insulin-induced uptake observed in untreated cells (154%).

In this study, nidulin not only stimulated 2-DG uptake in differentiated L6 myotubes, but also exerted additive effects when co-treated with insulin or metformin. These findings are crucial given the high rate of monotherapy failure in T2D management over time [25]. Nidulin also enhances both basal and insulin-stimulated 2-DG uptake in L6 myotubes with palmitate-induced insulin resistance. Palmitic acid is well-documented to impair insulin signaling by inducing mitochondrial dysfunction, thereby contributing to insulin resistance [26]. Collectively, our results support the potential of nidulin as an adjunctive therapeutic candidate for improving glucose metabolism in insulin-resistant conditions, warranting further mechanistic and in vivo studies.

### Nidulin-stimulated glucose uptake is slightly dependent on AMPK phosphorylation

**Fig. 3:**
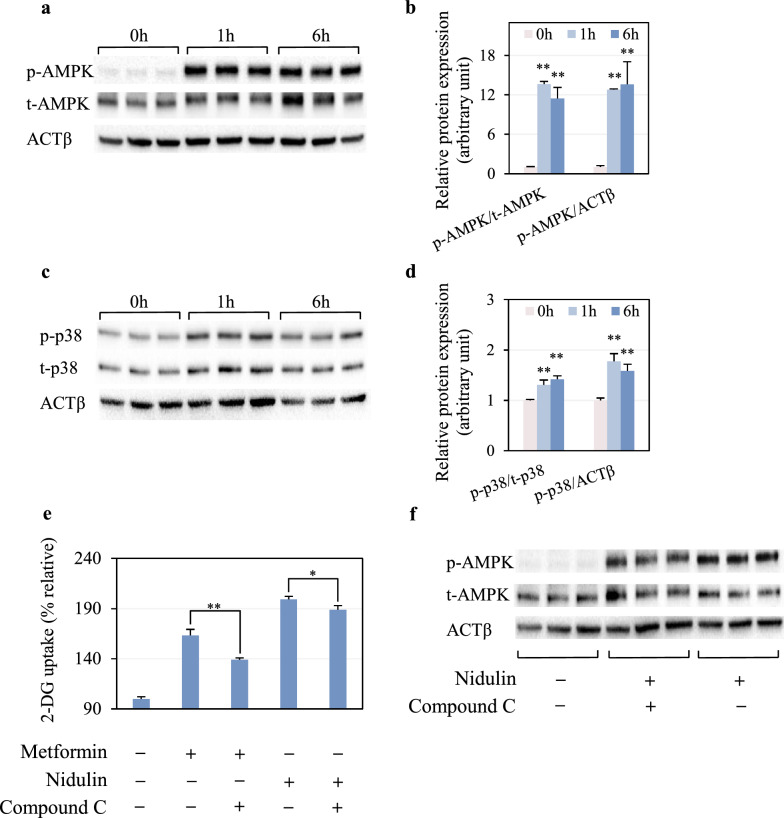
Effect of nidulin on the AMPK signaling pathway. **a** Immunoblot analysis of phosphorylated AMPK (p-AMPK), total AMPK (t-AMPK), and ACTβ as the loading control. **b** Quantification of p-AMPK levels normalized to t-AMPK and ACTβ. **c** Immunoblot analysis of phosphorylated p38 (p-p38), total p38 (t-p38), and ACTβ. **d** Quantification of p-p38 levels normalized to t-p38 and ACTβ. **e** Effect of AMPK inhibition on nidulin-stimulated 2-DG uptake. **f** Immunoblot analysis of p-AMPK, t-AMPK, and ACTβ in co-treatment of nidulin and compound C. L6 myotubes were treated with 20 µg/mL nidulin for 1 and 6 h. For inhibition studies, cells were pretreated with compound C for 1 h, then co-treated with nidulin and metformin as indicated for 3 h prior to 2-DG uptake assay and immunoblot analysis. DMSO, EtOH, and H_2_O were used as vehicle controls for nidulin, compound C, and metformin, respectively. Data are shown as relative 2-DG uptake (%) and expressed as mean ± S.D. (n = 3-4 per group). Similar results were obtained from the multiple experiments. Significant differences compared to the respective controls: *p < 0.05; **p < 0.01

To elucidate the mechanism of nidulin action, the involvement of AMPK and p38 signaling pathways in glucose uptake was investigated. As shown in Fig. 3a, nidulin profoundly increased the phosphorylation of AMPK. The phosphorylated (p-) AMPK to total (t-) AMPK ratios were elevated by 13.64-fold and 11.43-fold at 1 and 6 h treatment, respectively, compared to the untreated control (Fig. 3b). Similar trends were observed when normalized to ACTβ, indicating robust activation of AMPK by nidulin. In parallel, nidulin also induced phosphorylation of p38 (Fig. 3c). After 1 h of treatment, the p-p38/t-p38 and p-p38/ACTβ ratios increased by 1.31-fold and 1.78-fold, respectively (Fig. 3d). Although these increases persisted after 6 h, the magnitude of p38 activation was much less than that of AMPK activation. Because basal phosphorylation of AMPK and p38 could fluctuate over time, we additionally measured untreated cells at 0, 1, and 6 h. Only minimal to no changes in p-AMPK and p-p38 were detected, confirming that the robust activation observed was specifically induced by nidulin (Fig. S1). To assess the functional contribution of AMPK to nidulin-stimulated glucose uptake, cells were pretreated with compound C, an AMPK inhibitor. As expected, compound C reduced metformin-induced 2-DG uptake by ~ 40% (Fig. 3e), validating its inhibitory effect. However, the same treatment only modestly attenuated nidulin-stimulated 2-DG uptake, from 199 to 189% (a ~ 10% reduction). Western blotting further confirmed that compound C blocked nidulin-induced p-AMPK (Fig. 3f). Notably, despite clear inhibition of p-AMPK, compound C had only a marginal effect on nidulin-stimulated glucose uptake.

In insulin-responsive tissues, glucose uptake is primarily regulated by GLUT4 translocation to the plasma membrane via either the insulin-dependent or AMPK-mediated pathway. During exercise or muscle contraction, the increased AMP/ADP-to-ATP ratio activates AMPK which in turn promotes GLUT4 translocation [27]. p38, acting downstream of AMPK, also contributes to glucose uptake by enhancing GLUT4 trafficking [28]. Interestingly, nidulin treatment induced phosphorylation of both AMPK and p38. However, inhibition of AMPK phosphorylation using compound C had minimal impact on nidulin-stimulated glucose uptake, suggesting that the AMPK activation is not the primary mechanism through which nidulin exerts its effects. This points to the likely involvement of additional or alternative signaling pathways mediating the effect of nidulin.

### Nidulin-stimulated glucose uptake is regulated by the insulin signaling pathway

**Fig. 4:**
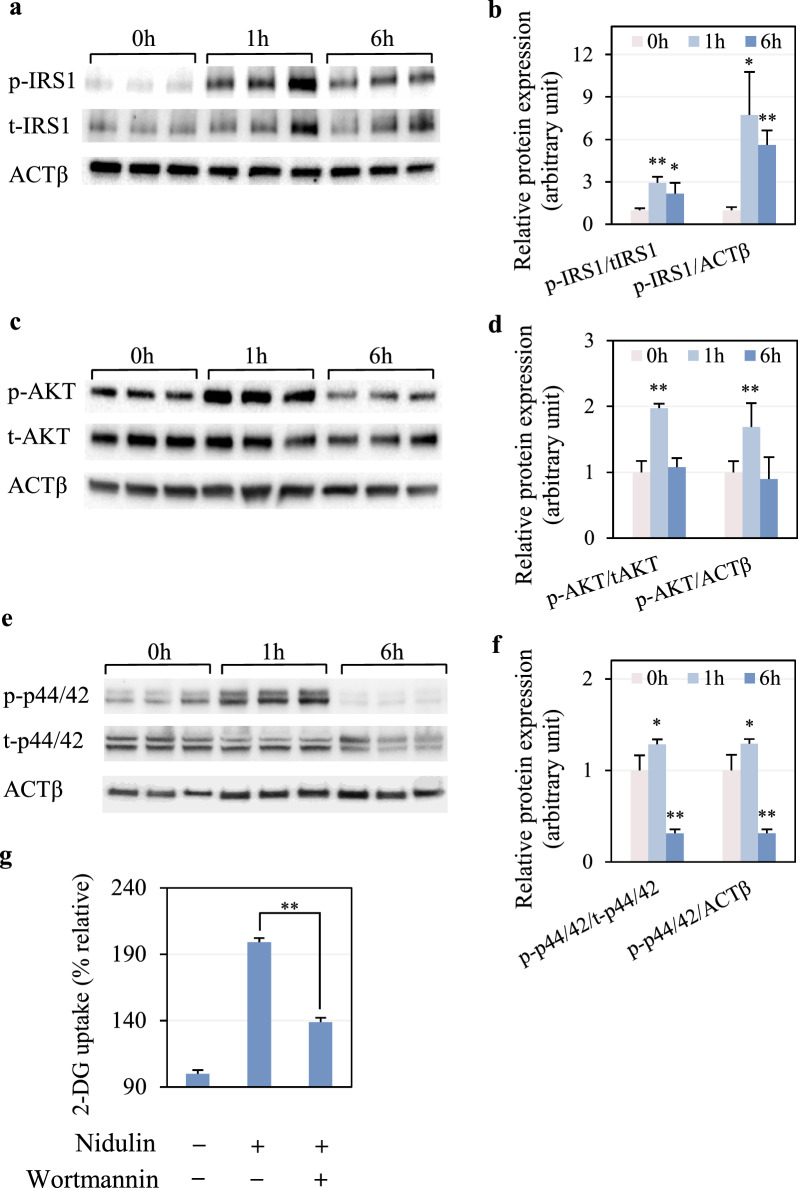
Effect of nidulin on the insulin signaling pathway. **a** Immunoblot analysis of phosphorylated IRS1 (p-IRS1), total IRS1 (t-IRS1), and ACTβ. **b** Quantification of p-IRS1 levels normalized to t-IRS1 and ACTβ. **c** Immunoblot analysis of phosphorylated AKT (p-AKT), total AKT (t-AKT), and ACTβ. **d** Quantification of p-AKT levels normalized to t-AKT and ACTβ. **e** Immunoblot analysis of phosphorylated p44/42 (p-p44/42), total p44/42 (t-p44/42), and ACTβ. **f** Quantification of p-p44/42 levels normalized to t-p44/42 and ACTβ. **g** Effect of PI3K/AKT inhibition on nidulin-stimulated glucose uptake. L6 myotubes were treated with 20 µg/mL nidulin for 1 and 6 h. For inhibition assays, cells were pretreated with wortmannin for 1 h, then co-treated with nidulin for 3 h prior to performing the 2-DG uptake assay. DMSO and EtOH were used as vehicle controls for nidulin and wortmannin, respectively. Data are presented as relative 2-DG uptake (%) normalized to untreated controls and expressed as mean ± S.D. (n = 3-4 per group). Similar results were obtained from the multiple experiments. Significant differences compared to the respective controls: *p < 0.05; **p < 0.01

To determine whether nidulin promotes glucose uptake through the insulin signaling cascade, the activation status of key signaling proteins, including IRS1, AKT, and p44/42, was examined following nidulin treatment at 1 and 6 h. As shown in Fig. 4a, nidulin significantly increased IRS1 phosphorylation. The ratio of p-IRS1 to t-IRS1 rose by 2.93-fold at 1 h and 2.16-fold at 6 h, relative to the untreated control (Fig. 4b). Comparable results were observed when normalized to ACTβ, indicating sustained IRS1 activation. Nidulin also stimulated AKT phosphorylation (Fig. 4c). At 1 h, p-AKT levels were significantly increased both normalized to t-AKT and ACTβ (Fig. 4d). Nidulin-stimulated p-AKT was enhanced distinctly up to 4 h (Fig. S2). However, this activation was transient, returning to baseline by 6 h. Similarly, p-p44/42 was stimulated by nidulin treatment (Fig. 4e). After 1 h, the ratio of p-p44/42 to t-p44/42 and ACTβ both increased by 1.29 folds, but diminished by 6 h, paralleling the AKT response (Fig. 4f). One may argue that the basal levels of p-p44/42 could be dynamic in the untreated conditions, but no change in p-p44/42 was observed in non-treatment among 0, 1, and 6 h (Fig. S3). To confirm the functional role of insulin signaling in nidulin-stimulated glucose uptake, cells were pretreated with wortmannin, a PI3K/AKT pathway inhibitor. As shown in Fig. 4g, wortmannin significantly attenuated nidulin-induced 2-DG uptake, reducing uptake from 199 to 138% compared to control.

Insulin-dependent glucose uptake is primarily mediated by the PI3K/AKT signaling pathway. Upon activation of IR, IRS is phosphorylated, initiating a signaling cascade through PI3K and AKT that culminates in GLUT4 translocation to the plasma membrane. Although p44/42 is not directly downstream of the PI3K/AKT axis, insulin has been shown to induce its phosphorylation, and this pathway can also contribute to the regulation of GLUT4 function [29]. In this study, we found that nidulin-stimulated glucose uptake is primarily mediated by the PI3K/AKT pathway. Nidulin enhanced the phosphorylation of IRS1, AKT, and p44/42, supporting its role in activating insulin-like signaling. Notably, inhibition of PI3K activity using wortmannin significantly reduced nidulin-induced glucose uptake. These findings suggest that nidulin promotes glucose uptake through the PI3K/AKT-dependent insulin signaling pathway.

### GLUT4 and GLUT1 expression and translocation are enhanced by nidulin

**Fig. 5:**
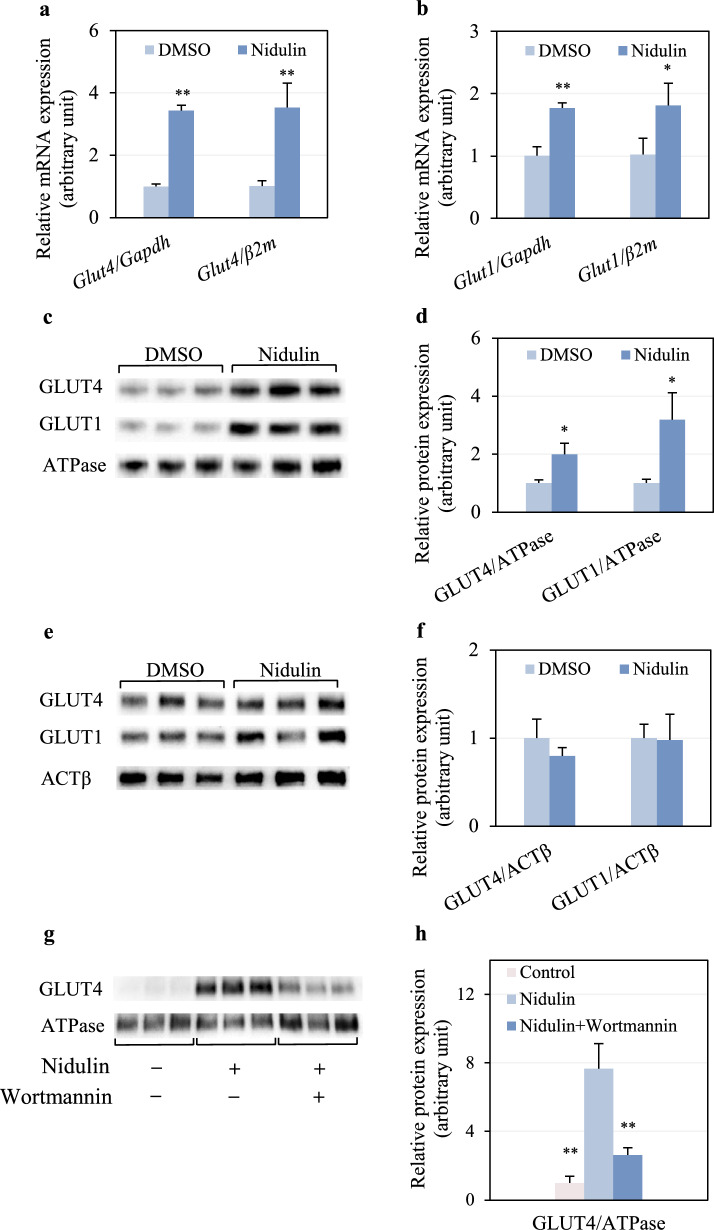
Stimulatory role of nidulin in GLUT4 and GLUT1 mRNA expression and translocation. **a** mRNA expression levels of GLUT4 normalized to GAPDH and β2M. **b** mRNA expression levels of GLUT1 normalized to GAPDH and β2M. **c** Immunoblot analysis of GLUT4, GLUT1, and Na⁺/K⁺ ATPase (ATPase) in the plasma membrane fraction. **d** Densitometric analysis of membrane-associated GLUT4 and GLUT1 normalized to ATPase. **e** Immunoblot analysis of GLUT4, GLUT1, and ACTβ in the cytosolic fraction. **f** Densitometric analysis of cytosolic GLUT4 and GLUT1 normalized to ACTβ. **g** Immunoblot analysis of membrane GLUT4 and ATPase in the presence of wortmannin. **h** Densitometric analysis of membrane-associated GLUT4 normalized to ATPase. L6 myotubes were treated with 20 µg/mL nidulin for 3, 9, and 16 h for inhibition assays, gene expression, and protein analysis, respectively. DMSO and EtOH were used as vehicle controls for nidulin and wortmannin, respectively. Gene expression was analyzed by qPCR using the 2^−ΔΔCT^ method. Membrane and cytosolic proteins were fractionated and detected via immunoblotting. Data represent mean ± S.D. (n = 3-4 per group). Similar results were obtained from the multiple experiments. Significant differences compared to the respective controls: *p < 0.05; **p < 0.01

To investigate whether nidulin enhances glucose uptake by modulating glucose transporter expression and localization, the mRNA and protein levels of GLUT4 and GLUT1 were assessed. As shown in Fig. 5a, nidulin treatment significantly upregulated the mRNA expression of both *Glut4* and *Glut1*. qPCR analysis revealed a 3.42- and 3.53-fold increase in *Glut4* expression when normalized to *Gapdh* and *β2m*, respectively, compared to the untreated control. Also, *Glut1* expression increased by 1.77- and 1.81-fold relative to *Gapdh* and *β2m*, respectively (Fig. 5b). As mentioned previously, GLUT4 is responsible for insulin stimulated-glucose uptake, while GLUT1 maintains basal glucose uptake. Increased *Glut4* expression in insulin-responsive tissues is a well-established therapeutic approach for improving insulin sensitivity and glycemic control [30, 31]. While nidulin enhances *Glut4* and *Glut1* expression levels, these transcriptional upregulations may represent an additional mechanism by which nidulin improves glucose uptake and mitigates insulin resistance.

To determine the subcellular localization of GLUT4 and GLUT1 proteins, plasma membrane and cytosolic fractions were subjected to immunoblot analysis. Nidulin treatment notably increased the levels of both transporters in the plasma membrane fraction (Fig. 5c). In particular, membrane-localized GLUT4 and GLUT1 levels, normalized to Na⁺/K⁺ ATPase, were 2.00- and 3.19-fold greater, respectively, than in the control group (Fig. 5d). In contrast, the cytosolic levels of GLUT4 and GLUT1 remained unchanged upon nidulin treatment, with relative expression levels of 0.80-fold and 0.98-fold, respectively, compared to the control (Fig. 5e, f). These results demonstrate that nidulin not only increases the transcription of *Glut4* and *Glut1*, but also facilitates their translocation to the plasma membrane, reinforcing its role in enhancing glucose uptake in myotubes. In insulin-responsive tissues, glucose uptake is primarily regulated by GLUT4 translocation to the plasma membrane via either the insulin-dependent or AMPK-mediated pathway. GLUT4 facilitates insulin- and exercise-stimulated glucose uptake, whereas basal glucose uptake is predominantly mediated by GLUT1. Our findings demonstrated that nidulin stimulates GLUT4 translocation to the plasma membrane. We also observed an increase in GLUT1 levels, but this is not unexpected, as GLUT4 and GLUT1 are known to colocalize, a phenomenon previously reported in insulin-treated myotubes [32]. As nidulin-stimulated glucose uptake is profoundly regulated through the PI3K/AKT pathway via GLUT4 translocation, future studies need to examine downstream Akt targets such as AS160 to further substantiate the mechanism of GLUT4 regulation by nidulin. In addition, this study investigated how inhibition of the PI3K/AKT pathway altered nidulin-mediated GLUT4 translocation. As shown in Fig. 5g, wortmannin declined the levels of nidulin-driven GLUT4 translocation to the plasma membrane. The ratio of the membrane-localized GLUT4 normalized to the Na⁺/K⁺ ATPase levels was 7.67-, and only 2.63-fold greater, in nidulin treatment alone and nidulin and wortmannin co-treatment, respectively, compared to the untreated controls. This evidence validates the effect of nidulin on GLUT4 translocation via the PI3K/AKT pathway responsible for glucose uptake in L6 myotubes.

### Nidulin modulates redox sensing and Ca^2+^ distribution similar to insulin

**Fig. 6:**
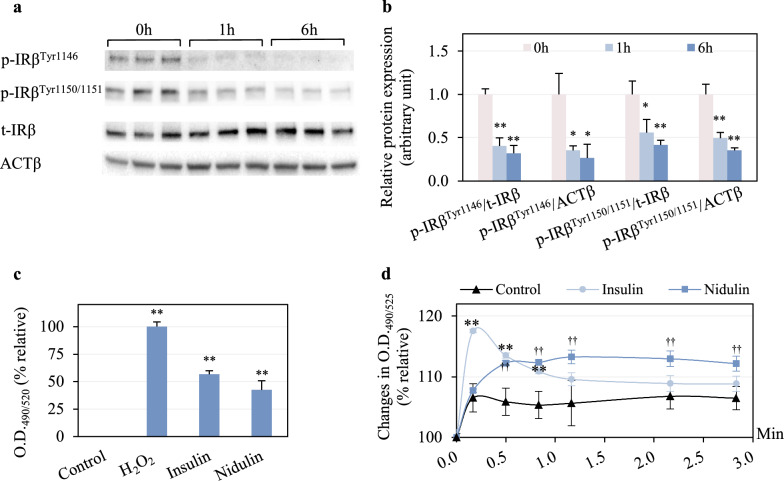
Upstream insulin signaling responses to nidulin. **a** Immunoblot analysis of phosphorylated insulin receptor β subunit at Ser307 (p-IRβ^Ser307^) and Tyr1150/1151 (p-IRβ^Tyr1150/1151^), total IRβ (t-IRβ), and ACTβ. **b** Quantitative analysis of protein bands in A. L6 myotubes were treated with 20 µg/mL nidulin for 1 and 6 h. **c** Intracellular H_2_O_2_ levels detected using AbGreen. Cells were treated with 100 µM H_2_O_2_ and 10 nM insulin for 20 min, and 20 µg/mL nidulin for 1 h. Fluorescence detection was measured at O.D._490/520_. and presented as relative fluorescence intensity (%) compared to 100 µM H₂O₂. **d** Cytosolic free Ca^2^⁺ content measured by Fluo-8. Cells were preloaded with the dye, followed by treatment with 100 nM insulin or 20 µg/mL nidulin. Fluorescence emission was recorded at O.D._490/525_ every 30 s for 3 min. Data represent relative changes (%) in fluorescence intensity. DMSO served as the vehicle control. Results are shown as mean ± S.D. (n = 3–5 per group). Similar results were obtained from the multiple experiments. Significant differences compared to control: ††*p* < 0.01 for nidulin; **p < 0.01 for insulin

To investigate how nidulin activates upstream insulin signaling pathways leading to AKT-mediated glucose uptake, IR phosphorylation was first examined. As shown in Fig. 6a, nidulin treatment did not promote p-insulin receptor β subunit (IRβ)^Tyr1146^ and p-IRβ^Tyr1150/1151^. In fact, phosphorylation at both sites was reduced. After 1 h of treatment, the relative abundance of p-IRβ^Tyr1146^ normalized to t-IRβ and ACTβ decreased to 0.40- and 0.38-fold, respectively, compared to the control (Fig. 6b). These reductions were sustained at 6 h. A similar pattern was observed for IRβ^Tyr1150/1151^ phosphorylation.

In insulin-dependent GLUT4 translocation, a critical early event is the phosphorylation of IR. Upon insulin binding to the IR α-subunit, a conformational change induces autophosphorylation of key tyrosine residues, including Tyr1146, Tyr1150, and Tyr1151, on the β-subunit, which is essential for activating the receptor’s intrinsic tyrosine kinase activity [33]. Once activated, the IR phosphorylates downstream targets, including IRS, thereby initiating the insulin signaling cascade [34]. In insulin-resistant states, both autophosphorylation and kinase activity of IR are often impaired, and total IR protein levels may be reduced [35, 36]. Given these defects, the identification of small molecules that activate the IR has attracted substantial interest [37]. For instance, demethylasterriquinone B1, a fungal metabolite from *Pseudomassaria*, directly induces phosphorylation of the IR β-subunit and functions as an insulin mimetic [38, 39]. In contrast, although nidulin significantly promotes glucose uptake in palmitic acid-induced insulin-resistant myotubes and stimulates GLUT4 translocation, our data suggest that IR is not its primary molecular target. Specifically, nidulin treatment reduced IR phosphorylation at Tyr1146, Tyr1150, and Tyr1151 to levels below those of untreated controls. One possible explanation is that IRS-1, once activated, may exert negative feedback on IR autophosphorylation [40].

The study next evaluated redox signaling and intracellular calcium mobilization which are auxiliary mechanisms known to modulate insulin signaling. As expected, insulin treatment significantly increased intracellular H₂O₂ levels, showing a 57% rise compared to both untreated and H₂O₂-treated controls (Fig. 6c). Nidulin treatment also elevated H₂O₂ levels, with a 43% increase relative to controls. In addition, insulin-induced intracellular Ca^2^⁺ mobilization was confirmed (Fig. 6d). A rapid increase in intracellular Ca^2^⁺ content was observed in 0.2 min of insulin treatment, followed by a decline to basal levels. Interestingly, nidulin also triggered a rise in intracellular Ca^2^⁺, though with a delayed onset at 0.5 min. The elevated Ca^2^⁺ levels persisted until 2.8 min, indicating a different kinetic profile from insulin.

Non-canonical insulin signaling pathways, particularly those involving redox regulation and intracellular Ca^2^⁺ signaling, may play a more prominent role [9, 41]. Beyond IR activation, insulin is known to elevate intracellular H₂O₂ and Ca^2^⁺ levels, which facilitate GLUT4 translocation in skeletal muscle. Specifically, insulin induces membrane translocation of the NADPH oxidase subunit p47, resulting in increased H₂O₂ production and Ca^2^⁺ mobilization [41]. Although chronic oxidative stress and sustained exposure to exogenous H₂O₂ have been shown to impair insulin-stimulated glucose uptake [42], the role of H₂O₂ in insulin signaling is paradoxical. Insulin itself stimulates H₂O₂ production, which facilitates GLUT4 translocation, underscoring the context-dependent nature of reactive oxygen species (ROS) in glucose metabolism [43]. Specifically, low concentrations of H₂O₂ (< 10 μM) can enhance AKT phosphorylation and potentiate insulin signaling, whereas higher concentrations (> 25 μM) disrupt signal transduction [44]. Moreover, moderate H₂O₂ accumulation such as that resulting from glutathione peroxidase 1 deficiency has been shown to improve insulin sensitivity in high-fat diet-fed mice [45]. In contrast, excessive mitochondrial H₂O₂ production has been causally linked to insulin resistance in both rodent models and humans [46]. These findings highlight the dual and concentration-dependent roles of ROS in modulating insulin-mediated glucose uptake, providing important context for the redox-sensitive mechanisms underlying the effects of nidulin [47]. In summary, our findings suggest that nidulin does not activate IR directly, but initiates glucose uptake through alternative upstream mechanisms.

### Inhibition of redox and Ca^2+^ changes abolishes nidulin-stimulated AKT and 2-DG uptake

**Fig. 7:**
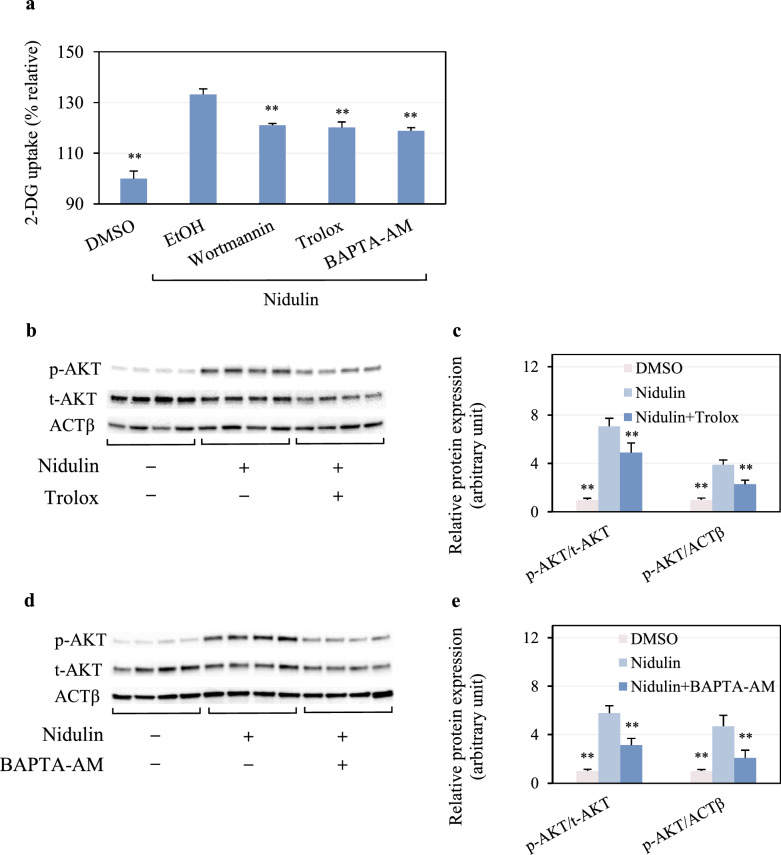
Modulation of redox and Ca^2^⁺ signaling in nidulin-induced glucose uptake and AKT activation. **a** 2-DG uptake in L6 myotubes treated with 20 µg/mL nidulin for 3 h in the presence or absence of 1 mM Trolox, 10 µM BAPTA-AM, or 100 nM wortmannin. **b** and **c** Immunoblot analysis of phosphorylated AKT (p-AKT) and total AKT (t-AKT) protein levels following nidulin treatment with or without Trolox or BAPTA-AM, respectively. **d** and **e** Densitometric quantification of p-AKT normalized to t-AKT and ACTβ. L6 myotubes were pretreated with inhibitors for 1 h prior to co-treatment with nidulin in the differentiation medium with 0.5% horse serum. The 2-DG uptake assay and protein analysis were performed. Data are shown as relative 2-DG uptake (%) and expressed as mean ± S.D. (n = 4 per group). Similar results were obtained from the multiple experiments. **p < 0.01 compared to the nidulin-treated group

To validate the involvement of redox and Ca^2^⁺ signaling in nidulin-stimulated glucose uptake, specific inhibitors were utilized. Trolox, a ROS scavenger, and BAPTA-AM, an intracellular Ca^2^⁺ chelator, were employed to neutralize H₂O₂ and block Ca^2^⁺ mobilization, respectively. As shown in Fig. 7a, both Trolox and BAPTA-AM significantly suppressed nidulin-induced 2-DG uptake, with effects comparable to that of wortmannin. To investigate whether these inhibitory effects were linked to the insulin signaling pathway, the phosphorylation status of AKT was assessed. As expected, nidulin alone markedly increased AKT phosphorylation (Fig. 7b). However, co-treatment with Trolox substantially reduced p-AKT levels. The levels of p-AKT normalized to that of t-AKT and ACTβ were a 0.69- and 0.59-fold decrease, respectively, in the presence of Trolox compared to nidulin treatment (Fig. 7c). The reduction of p-AKT levels was similarly observed in co-treatment of nidulin and BAPTA-AM compared to nidulin alone (Fig. 7d). As shown in Fig. 7e, p-AKT levels normalized to t-AKT and ACTβ were 0.54- and 0.44-fold lower, respectively, in co-treatment than in nidulin treatment alone. These findings support the notion that nidulin activates glucose uptake via redox-sensitive and Ca^2^⁺-dependent mechanisms that converge on the insulin-responsive PI3K/AKT signaling pathway.

This study demonstrated that nidulin increased intracellular H₂O₂ and Ca^2^⁺ levels. Interruption of either H₂O₂ or Ca^2^⁺ signaling has been shown to impair insulin-stimulated GLUT4 translocation. Nidulin treatment increases intracellular H₂O₂ and Ca^2^⁺ levels, both of which are known to act as secondary messengers that modulate insulin signaling. Although the precise molecular events by which nidulin regulates these signals upstream of AKT remain to be clarified, the observed redox and Ca^2^⁺ dynamics suggest a potential mechanism whereby nidulin facilitates AKT phosphorylation and GLUT4 translocation [9, 41]. These findings add a new dimension to the understanding of fungal metabolites in metabolic regulation. We acknowledge that further mechanistic studies are needed to delineate how nidulin directly influences redox balance and Ca^2^⁺ handling and how these changes are integrated into the IRS/AKT signaling cascade. Nevertheless, by establishing this link, our study provides a valuable foundation for future work aimed at unraveling the hypoglycemic potential of nidulin. These findings suggest that nidulin enhances GLUT4 translocation associated with ROS- and Ca^2^⁺-dependent pathways and acts, at least in part, via PI3K/AKT activation.

## Conclusions

This study demonstrates that nidulin, a trichlorinated fungal depsidone, significantly enhances glucose uptake in L6 myotubes under both insulin-sensitive and insulin-resistant conditions. Nidulin promotes GLUT4 translocation primarily via activation of the PI3K/AKT signaling pathway. In particular, nidulin-induced redox and Ca^2^⁺ signaling act as upstream modulators of this pathway, partially mimicking certain aspects of insulin action despite bypassing insulin receptor activation. Additionally, this fungal metabolite upregulates *Glut4* and *Glut1* expression and restores glucose uptake impaired by palmitate-induced insulin resistance. It is important to evaluate the glycemic control effects of nidulin in animal models. Although in vivo studies on depsidone metabolites remain limited, previous reports have demonstrated well tolerability of these compounds in animals [48, 49]. In line with these findings, nidulin emerges as a promising candidate for further investigation in models of insulin resistance and T2D. This study suggests that nidulin partially mimics insulin action holding potential as a novel insulin-sensitizing agent, highlight the potential of nidulin as an adjunctive therapy for insulin resistance and T2D.

## Experimental procedures

### Nidulin induction and purification

*A. unguis* ATCC 10032 was cultured in potato dextrose broth (PDB; 0.04% potato starch (Difco), 0.2% dextrose (Difco), and 0.02% NaCl (Sigma-Aldrich)) at 25 °C for 40 days. Nidulin was extracted from the mycelium and culture medium as previously described [16]. Briefly, the harvested mycelium was incubated with an equal volume of methanol (MeOH) (Sigma-Aldrich) relative to the culture broth at 25 °C for 4 days. The resulting solution was filtered to collect the nidulin-containing solvent. To separate phases, hexane and water were added to the collected solution in a 1:0.4:1 ratio. The MeOH–water bottom phase was concentrated by evaporation, diluted with water, and then extracted with ethyl acetate (EtOAc) (Sigma-Aldrich). The culture broth was extracted twice with an equal volume of EtOAc. The EtOAc fractions were pooled, concentrated under vacuum, and subjected to silica gel column chromatography (6.5 × 17 cm, dichloromethane/hexane, gradient from 40:60 to 100:0). The nidulin-containing fractions were further purified by preparative HPLC using a reversed-phase column (Shiseido CAPCELL PAK C18, 20 × 250 mm, 5 μm; MeCN/water = 65:35, flow rate 12 mL/min). Compound identification was determined using ^1^H NMR spectroscopy, high-resolution electrospray ionization mass spectrometry (HRESIMS), and its purity was determined to be > 99% by HPLC analysis. For subsequent experiments, nidulin was dissolved in dimethyl sulfoxide (DMSO) (10 mg/mL) and stored at −20 °C.

### Cell culture and differentiation

L6 myoblasts were obtained from ATCC and cultured as previously described [50]. Cells were maintained in DMEM (Cytiva) supplemented with 4.5 g/L glucose, 10% fetal bovine serum (FBS, Cytiva), 50 U/mL penicillin, and 50 µg/mL streptomycin (Cytiva) at 37 °C in a 5% CO₂ atmosphere. For differentiation into myotubes, 6 × 10^4^ cells/mL were seeded and grown to 80% confluence. The medium was then replaced with MEMα (Cytiva) containing 2% HS (Cytiva). The cells were cultured for 6 days, with medium changes every 2 days.

### Cell treatment

L6 myotubes were treated with nidulin (10, 20, or 30 µg/mL) in medium containing either 2% or 0.5% HS at 37 °C and 5% CO₂ for 18 h to determine a non-cytotoxic range of nidulin. For dose-dependent studies, L6 myotubes were treated with nidulin (1.25, 2.5, 5, 10, and 20 µg/mL) in medium for 16 h. For time-course experiments, cells were incubated with 20 µg/mL nidulin for 3, 6, 12, or 16 h. Unless otherwise specified, subsequent experiments were pre-incubated in differentiation medium with 0.5% HS for 3 h and treated with 20 µg/mL nidulin at 37 °C with 5% CO_2_. DMSO was used as the vehicle control. 10 or 100 nM insulin as indicated, and 1 mM metformin were used for cell treatment for 30 min and 16 h, respectively. For signaling inhibition assays, cells were pretreated with 20 µM compound C (Sigma-Aldrich) or 0.5 µM wortmannin (Sigma-Aldrich) for 1 h, and then co-treated with nidulin or 1 mM metformin (Sigma Aldrich) for 16 h. EtOH was used as the vehicle control of the pharmacological inhibitors where applicable. For antioxidant and calcium chelation experiments, cells were pretreated with 2.5 µM Trolox (Sigma-Aldrich) or 10 µM BAPTA-AM (Abcam) for 1 h, followed by co-incubation with nidulin for 3 h in the differentiation medium (0.5% HS). EtOH and DMSO served as the vehicle control for Trolox and BAPTA-AM, respectively.

### Cell cytotoxicity assay

Cell viability was assessed using a fluorometric green assay kit (Abcam) according to the manufacturer’s instructions. Viable cells were stained and fluorescence was measured at Ex/Em = 490/520 nm using a SPARK Multimode Microplate Reader (TECAN).

### 2-[^3^H]-deoxy-glucose (2-DG) uptake assay

Following treatment, cells were washed twice with PBS and incubated in Krebs–Ringer HEPES (KRH) buffer at 37 °C for 30 min. The buffer was then replaced with KRH containing 0.3 µM 2-DG (PerkinElmer) and 150 µM D-glucose. Uptake was allowed to proceed at 37 °C for 15 min, after which the reaction was terminated by washing the cells three times with ice-cold PBS supplemented with 20 mM D-glucose. Cells were lysed in 0.1 M NaOH, and intracellular 2-DG levels were quantified using a MicroBeta2 scintillation counter (PerkinElmer). Total protein content was measured by BCA protein assay (Merck), and 2-DG uptake was normalized to protein concentration.

### Palmitic acid-induced insulin resistance

A 100 mM sodium palmitate (Sigma-Aldrich) stock solution was prepared in 70% ethanol and stored at −20 °C. For induction of insulin resistance, 125 µM sodium palmitate was freshly prepared in differentiation medium (2% HS) containing 12.5 µM fatty acid-free BSA (Gold Biotechnology), and stirred at 45 °C for 30 min. L6 myotubes were treated with the palmitate-containing medium at 37 °C for 16 h. A 70% ethanol solution was used as the vehicle control. Where indicated, nidulin was co-incubated with palmitate during the treatment period. For insulin-stimulated conditions, cells were treated with 100 nM insulin (Sigma-Aldrich) for 30 min prior to the 2-DG uptake assay.

### RNA isolation and gene expression analysis

Cells were washed with ice-cold PBS and lysed in GENEzol^™^ reagent (Geneaid). Total RNA was extracted according to the manufacturer’s instructions. Reverse transcription was performed using the iScript^™^ cDNA Synthesis Kit (Bio-Rad) to generate cDNA. qPCR was conducted using a CFX384 Real-Time PCR Detection System (Bio-Rad), with melting curve analysis to verify primer specificity. Reactions were performed in technical duplicates using qPCRBIO SYGreen Mix Lo-ROX (PCR Biosystems). Gene-specific primers were used for *Glut4*, *Glut1*, *Gapdh*, and *β2m* [51]. Relative mRNA expression levels of *Glut4* and *Glut1* were normalized to the geometric mean of *Gapdh* and *β2m* and calculated using the 2^−ΔΔCT^ method.

### Total protein isolation

Cells were rinsed with ice-cold PBS and lysed using M-PER reagent (Thermo Fisher Scientific) supplemented with protease inhibitors (cOmplete^™^ ULTRA tablets, Roche), kinase inhibitors, and phosphatase inhibitors [52]. Lysates were centrifuged at 12,000 × *g* for 5 min at 4 °C, and the resulting supernatant containing total protein was collected. Protein concentrations were measured using a BCA protein assay, and samples were stored at − 80 °C for subsequent analysis.

### Cytosol and plasma membrane protein isolation

Cells were washed with ice-cold PBS, harvested, and centrifuged at 12,000 rpm for 10 min at 4 °C. Cytosolic and plasma membrane fractions were isolated using a plasma membrane protein extraction kit (Abcam) following the manufacturer’s protocol. Protein concentrations were determined using the BCA protein assay.

### Immunoblot analysis

Protein samples were resolved on 4–20% Mini-PROTEAN^®^ TGX^™^ gels (Bio-Rad) and transferred to PVDF membranes (Bio-Rad) using the Trans-Blot Turbo Transfer System (Bio-Rad). Membranes were blocked with 5% skim milk (Difco) in PBST (PBS containing 0.1% Tween-20). The following primary antibodies (Cell Signaling Technology) were used: p-AMPK (Thr172), t-AMPK, p-p38 (Thr180/Tyr182), t-p38, p-IRS1 (Tyr895), t-IRS1, p-AKT (Ser473), t-AKT, p-p44/42 (Thr202/Tyr204), t-p44/42, p-IRβ (Tyr1146 and Tyr1150/1151), t-IRβ, GLUT1, GLUT4, Na⁺/K⁺ ATPase, and ACTβ. Membranes were incubated with primary antibodies diluted in PBST overnight at 4 °C. HRP-conjugated secondary antibodies (Cell Signaling Technology) were applied in blocking solution for 1 h at room temperature. Signals were developed using Immobilon^®^ Forte (Millipore) or SuperSignal^™^ West Femto (Thermo Fisher Scientific) chemiluminescent substrates and visualized using the ChemiDoc XRS + System (Bio-Rad). Band intensities were quantified using Image Lab software (Bio-Rad).

### Intracellular H_2_O_2_ and Ca^2+^ detection

Intracellular H₂O₂ and Ca^2^⁺ levels were assessed using a cell-based hydrogen peroxide assay kit (Abcam) and Fluo-8 calcium flux assay kit (Abcam), respectively. Assays were conducted according to the manufacturer’s instructions. H₂O₂ and Ca^2^⁺ were detected using AbGreen and Fluo-8 indicators, respectively, and fluorescence was measured using a SPARK Multimode Microplate Reader (TECAN) at Ex/Em = 490/520 nm.

### Statistical analysis

All experiments were performed in at least triplicate. Data are presented as mean ± standard deviation (S.D.). Statistical comparisons between groups were conducted using the Student’s *t*-test. Differences were considered statistically significant at *p* < 0.05.

## Supplementary Information

Supplementary material 1.

## Data Availability

All data are presented in this article. No additional data were generated or analyzed.
