# Supplementary material for: Nidulin stimulates glucose uptake in myotubes through the IRS-AKT pathway and alters redox balance and intracellular calcium
Source: Nat Prod Bioprospect. 2025 Sep 11;15(1):63. doi: 10.1007/s13659-025-00546-3 (PMC12425880; doi:10.1007/s13659-025-00546-3)
Supplement: Supplementary file 1 — Supplementary material 1. [file 13659_2025_546_MOESM1_ESM.docx]

**Supporting Information**

**Nidulin stimulates glucose uptake in myotubes through the IRS-AKT pathway and alters redox balance and intracellular calcium**

Kanittha Chantarasakha^1^, Arunrat Yangchum^1^, Masahiko Isaka^1^, and Surapun Tepaamorndech^2^*

^1^National Center for Genetic Engineering and Biotechnology (BIOTEC), National Science and Technology Development Agency (NSTDA), 111 Thailand Science Park, Phahonyothin Road, Klong Luang, Pathumthani 12120, Thailand

^2^Department of Microbiology, Faculty of Medicine, Chulalongkorn University and King Chulalongkorn Memorial Hospital, Bangkok, 10330, Thailand

*Corresponding author

Surapun Tepaamorndech, Ph.D.

Email: surapun.t@chula.ac.th

**List of Supporting Information**

|  | Page |
| --- | --- |
| **Fig. S1** Immunoblot analysis of AMPK and P38 phosphorylation | 3 |
| **Fig. S2** Immunoblot analysis of AKT phosphorylation in nidulin treatment | 4 |
| **Fig. S3** Immunoblot analysis of P44/42 phosphorylation | 5 |

**Fig. S1** Immunoblot analysis of AMPK and P38 phosphorylation and ACTβ. **a** Protein levels of phosphorylated AMPK (p-AMPK), total AMPK (t-AMPK), and ACTβ as the loading control at 0, 1, and 6 h. **b** Quantification of p-AMPK levels normalized to t-AMPK and ACTβ. **c** Protein levels of phosphorylated p38 (p-p38), total p38 (t-p38), and ACTβ. **d** Quantification of p-p38 levels normalized to t-p38 and ACTβ. L6 myotubes were treated with DMSO as a vehicle for nidulin for 1 and 6 h. The cells were treated with 20 µg/mL nidulin for 1 h were loaded for comparisons. Data are expressed as mean ± S.D. (n = 3 per group). Comparison among time points was determined using ANOVA. N.S., not significant; ** *p* < 0.01.

**Fig. S2** Immunoblot analysis of AKT phosphorylation in nidulin treatment. Protein levels of phosphorylated AKT (p-AKT), and total AKT (t-AKT) as the loading control. L6 myotubes were treated with 20 µg/mL nidulin from 0.5 to 8 h as indicated.

**Fig. S3** Immunoblot analysis of P44/42 phosphorylation. **a** Protein levels of phosphorylated P44/42 (p-P44/42), total P44/42 (t-P44/42), and ACTβ as the loading control at 0, 1, and 6 h. **b** Quantification of p-P44/42 levels normalized to t-P44/42 and ACTβ. L6 myotubes were treated with DMSO as a vehicle for nidulin for 1 and 6 h. The cells treated with 20 µg/mL nidulin for 1 h were used for comparisons. Data are expressed as mean ± S.D. (n = 3 per group). Comparisons were determined using ANOVA. N.S., not significant.
